# Tongue papillitis and volatile sulfur compounds (VSC) values in a COVID-19 patient

**DOI:** 10.11604/pamj.2022.41.5.28915

**Published:** 2022-01-03

**Authors:** Cinzia Casu, Germano Orrù

**Affiliations:** 1 Department of Surgical Science, Oral Biotechnology Laboratory, University of Cagliari, Cagliari, Italy

**Keywords:** Tongue papillitis, oral manifestation, volatile sulfur compounds, COVID-19, tongue

## Image in medicine

A 30-year-old male patient in general good health came to my observation for a lingual checkup. The patient had tested positive for SARS-CoV-2 with a molecular swab about 1 month earlier, with mild symptoms of COVID-19: fever at 37.6°c for a day, mild sore throat. No alteration of flavors and odors. Upon physical examination, he showed excellent dento-periodontal health and very good oral hygiene. At the tip and lingual margins, there were asymptomatic red pinpoint spots, which the patient had noticed appearing from the first day of COVID-19 symptoms. A diagnosis of papillitis had been made and an examination with a gas chromatograph Oral Chroma™ (Oral Chroma™, Abimedical, Abilit Corp., Osaka, Japan) was performed to evaluate tongue Volatile Sulfur Compounds (VSC) values with the aim to intercept any gastric or respiratory problems. The report showed very high levels of (CH_3_)_2_S (735, when the maximum is 9) closely associated with gastric pathologies. An explanation of the phenomenon would lead to think that the SARS-CoV-2 virus had proliferated more in the gastric than in the respiratory field. The presence of papillitis in patients with COVID-19 has previously been documented in the scientific literature but has never been correlated with oral VSC values. Differential diagnosis includes: lingual irritation from savory or spicy foods, papillitis not linked to COVID-19 or mild alteration of the normal shape of the lingual mucosa in people who abuse alcohol.

**Figure 1:**
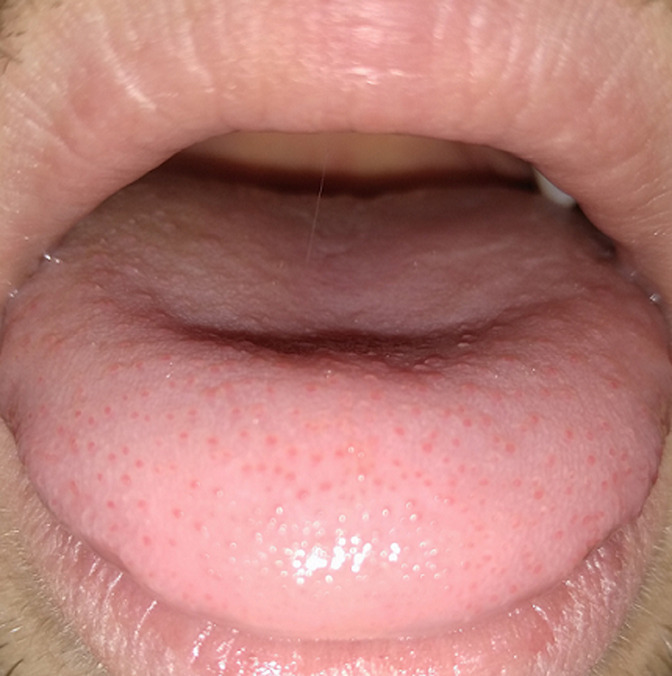
tongue papillitis in a COVID-19 patient that showed very high values of oral (CH_3_)_2_S

